# Delay in Seeking Medical Help following Transient Ischemic Attack (TIA) or “Mini-Stroke”: A Qualitative Study

**DOI:** 10.1371/journal.pone.0104434

**Published:** 2014-08-19

**Authors:** Jennifer Mc Sharry, Alison Baxter, Louise M. Wallace, Anthony Kenton, Andrew Turner, David P. French

**Affiliations:** 1 Health Behaviour Change Research Group, School of Psychology, National University of Ireland, Galway, Ireland; 2 Applied Research Centre in Health & Lifestyle Interventions, Coventry University, Coventry, United Kingdom; 3 University Hospitals Coventry and Warwickshire NHS Trust, Coventry, West Midlands, United Kingdom; 4 Manchester Centre for Health Psychology, University of Manchester, Manchester, United Kingdom; University of St Andrews, United Kingdom

## Abstract

**Background:**

Prompt treatment following Transient Ischemic Attack (TIA) can reduce the risk of subsequent stroke and disability. However, many patients delay in making contact with medical services. This study aimed to explore TIA patients' accounts of delay between symptom onset and contacting medical services including how decisions to contact services were made and the factors discussed in relation to delay.

**Methods:**

Twenty interviews were conducted with TIA patients in England. Using a previous systematic review as an initial framework, interview data were organised into categories of symptom recognition, presence of others and type of care sought. A thematic analysis was then conducted to explore descriptions of care-seeking relevant to each category.

**Results:**

Delay in contacting medical services varied from less than an hour to eight days. Awareness of typical stroke symptoms could lead to urgent action when more severe TIA symptoms were present but could lead to delay when experienced symptoms were less severe. The role of friends and family varied widely from deciding on and enacting care-seeking decisions to simply providing transport to the GP practice. When family or friends played a greater role, and both made and enacted care-seeking decisions, delays were often shorter, even when patients themselves failed to identify symptoms. Healthcare professionals also impacted on patients' care-seeking with greater delays in seeking further care for the same episode described when patients perceived a lack of urgency during initial healthcare interactions.

**Conclusions:**

This study provides new information on patients' decisions to contact medical services following TIA and identifies overlapping factors that can lead to delay in receiving appropriate treatment. While recognition of symptoms may contribute to delay in contacting medical services, additional factors, including full responsibility being taken by others and initial healthcare interactions, can over-ride or undermine the importance of patients' own identification of TIA.

## Introduction

Transient Ischemic Attack (TIA), also known as mini-stroke, warning stroke, or transient stroke, has traditionally been defined as a sudden neurological deficit with symptoms of less than 24 hours duration [1]. More recent definitions have moved away from the specification of a time cut-off for symptom duration; however the transient nature of TIA symptoms remains a key identifying factor [2], [3]. Although prevalence estimates vary by definition used, TIA is common with 2.8% of US adults reporting a physician diagnosis of TIA and a further 3.2% describing symptoms consistent with TIA [4].

TIA has been described as a warning sign for future stroke with the seven day, 30 day and 90 day risks of stroke following TIA estimated at 5.2%, 8% and 9.2% respectively [5], [6]. Studies have shown that urgent assessment and clinic treatment following TIA can decrease 6-month disability and result in an 80% reduction in subsequent stroke [7]–[9]. Despite the health benefits associated with early treatment, almost half of patients fail to seek medical attention within 24 hours of TIA [10].

There are several models that attempt to capture the reasons why patients delay in seeking medical help once symptoms occur [11]. One popular model [12] proposes that there are five distinct stages: (a) time for evaluation of somatic information as indicating illness, (b) time from deciding illness is present to seeking professional medical care, (c) time from this decision to making an appointment, (d) time from making an appointment to being seen by a medical professional, and (e) time from seeing a medical professional to obtaining treatment. Other authors [13] propose that in practice it is difficult to distinguish between (b) and (c), and that there are two main processes in determining patient help seeking behaviour: symptom appraisal and implementation of a response once a symptom has been appraised as indicating illness. The present study focuses on these two sources of delay in help seeking behaviour, rather than the final two stages, which are to do with the healthcare system.

These models of delay in seeking medical help have been used in a range of medical conditions including major stroke [11]. For example, previous quantitative research has demonstrated that making sense of symptoms is an important source of delay in reaching hospital following the onset of myocardial infarction [14]. When there is a mismatch between symptoms patients expect to indicate a heart attack (e.g. pain and collapse) and symptoms experienced (e.g. fever) delays in reaching hospital can be longer.

Evidence on delays in seeking medical help following TIA is more limited. A recent systematic review of quantitative studies investigating delay in seeking medical attention following TIA identified only one study [10] conducted in patients with TIA alone with most studies reporting on mixed TIA and stroke samples [15]. Drawing on the limited available evidence, Sprigg and colleagues identified a number of factors associated with delay in receiving medical attention following TIA: type of care sought, witness presence at symptom onset and symptom recognition [15].

The most consistent evidence identified was for type of care sought. Presentation at emergency services was associated with reduced delay across all reviewed studies even though the majority of TIA patients first contacted their general practitioner (GP). Witness presence was investigated as a factor influencing care-seeking in two reviewed studies. Initial recognition of symptoms by friends or family was associated with reduced care-seeking delay in the two mixed sample studies reviewed; no studies were identified which explored the influence of witness presence on care-seeking in TIA patients alone. Finally, there was inconsistent evidence across studies of an association between patients' recognition of symptoms and care-seeking delay [15].

Overall, the existing quantitative evidence on factors influencing care-seeking delay in TIA patients is limited and inconsistent. Qualitative research can offer insight into complex behaviours and help in understanding inconsistencies in quantitative findings. Similar to the help-seeking literature more broadly, qualitative studies have focused on stroke rather than TIA. Two recent papers reported on decisions to seek care following stroke through interviews with a single sample of 19 stroke patients and 26 witnesses present at symptom onset [16], [17]. The first paper, focusing on the stroke witnesses alone, identified the use of rules of thumb, environmental context and resources, social influence, and beliefs about consequences as factors that influence decisions to seek care on behalf of patients [16]. The second paper analysed both patient and witness accounts and identified stroke severity, fear of stroke consequences, involving others and making sense of symptoms as influencing help-seeking decisions [17].

Although TIA and stroke share similarities, TIA patients differentiate their experience on the basis of the temporary nature of TIA and the expectation of full recovery [18]. TIA patients also describe symptom experiences which differ from those typically associated with a traditional stroke presentation [19]. The transient nature of TIA symptoms may impact on TIA recognition or on the role of others in care-seeking and has not previously been explored in a qualitative study with this population. In addition, the existing literature has not addressed which symptoms are expected and experienced by TIA patients and any potential relationships with delays in contacting medical services.

The aim of the current study was to explore delay between symptom onset and seeking medical care as described by a sample of patients diagnosed with TIA. Given the lack of evidence on others seeking care on behalf of TIA patients, we focused on patients' own experiences and their accounts of the role of friends and family members. Using the systematic review of Sprigg and colleagues [15] as a guide, we wished to explore how decisions to seek care were made, the length of delay, and the factors discussed by patients in relation to delays in contacting medical services.

## Methods

### Ethics Statement

Ethical approval was received from Warwickshire Research Ethics Committee (REC 09/H1211/89). All participants provided written informed prior to participation. The participant consent form is shown in Appendix S1.

### Procedure

Eligible patients had received a TIA diagnosis from a health care professional and were recruited by TIA nurses following attendance at a TIA hospital clinic. TIA nurses specialise in the care of TIA and stroke patients and participate in the running of TIA clinics at hospitals. Patients are referred to a TIA nurse either through their GP or through the hospital admissions department. TIA nurses from three participating hospitals in a single region of the English Midlands supplied an email list every week of all patients seen at their outpatient clinics in the preceding week who had experienced a recent TIA.

All patients gave permission for their details to be passed to the research team. AB then contacted patients by telephone, described the study and, where patients gave verbal consent, sent on a patient information sheet and arranged a time for interview. All interviews were conducted by AB. AB has a background in Health Psychology and had limited previous knowledge of TIA before conducting the interviews. Interviews were conducted face-to-face, digitally recorded and transcribed.

### Sample

TIA nurses identified 44 new TIA patients over a three month recruitment period in 2011. Of these, eight did not want to participate, two were judged not competent to give informed consent, and 14 did not respond to letters sent out and were not contactable. Those who agreed to participate did not differ from those who did not in terms of gender and postcode index of multiple deprivation scores. Recruitment continued until rich sufficient data to answer the research questions had been collected and no new significant insights were emerging from interviews [20].

Twenty patients were interviewed and family members were present in eight cases. All interviews were conducted in the participants' homes. Participants were aged between 45 and 89 years (mean = 71.8 years, SD = 12.1), all were white British and 12 were male. All participants had left education by the age of 19 years, with the majority leaving at 14 years (n = 8) or 15 years (n = 6) of age. Two participants had a previous history of TIA (two years and eight years previously), three participants had a previous history of stroke (two years, three years and 10 years previously), and fifteen participants had no previous history of TIA or stroke.

### Interview Schedule

A semi-structured interview schedule with prompts (see Appendix S2) was developed using an autobiographical approach, where patients were asked to recount what had happened to them during their TIA, what actions were taken and the reasons for these actions [21]. In addition, participants were asked to describe the symptoms they would have expected to indicate TIA or stroke prior to their own experience and the symptoms actually experienced during the TIA event. The interview schedule had previously been pilot-tested through telephone interviews.

### Analysis

Analysis was conducted primarily by JMS in collaboration with DF. JMS and DF are Health Psychologists with experience in conducting qualitative research. JMS had not been involved in the design of the study or in conducting the interviews and had no prior assumptions related to the data, other than previous experience of conducting health research. Initial analysis began with data immersion and successive readings of interview transcripts. Using the factors identified as impacting on TIA care-seeking in the systematic review of Sprigg and colleagues [15] as a guiding framework, interview data were grouped into categories of symptom recognition, presence of others and type of care sought. These categories were further divided into relevant sub-categories (e.g. other present, other not present) and the number of participants and length of delay within each sub-category recorded.

A thematic analysis [22] was then conducted to explore how care-seeking decisions and behaviours were described by participants within each sub-category. Initial low-level codes were applied to data and codes that reflected similar overarching ideas were grouped together. The initial categories and sub-categories applied to the data were modified and expanded on during this process and the new labels were applied to the data. The process of categorising and coding data was conducted by JMS and discussed in regular meetings with DF. The findings were also discussed with the other members of the multi-disciplinary research team. The software program NVivo10 (QSR International Pty Ltd, Doncaster, Victoria, Australia) was used to help with data organization and coding during analysis.

## Results

The interviews were typically 30 minutes long and ranged in length from 17 to 44 minutes. For 12 participants there was less than one hour delay in contacting medical services, four participants sought care within 24 hours and for four participants delays stretched to longer than a day. The themes identified in participants' descriptions of care-seeking are summarised below under the categories of symptom recognition, care-seeker, type of care sought and secondary delays and discussed with reference to delay in contacting medical services. Quotes are supplemented with information on participant gender, age, previous TIA or stroke, care-seeker and length of delay. For ease of reference, these characteristics are summarised for all participants in Table 1.

**Table 1:** Age, care-seeking delay, care-seeker, care sought and TIA recognition for all participants.

Participant	Age	Care-Seeking Delay	Care-Seeker	Care Sought	TIA Recognised
P20	85	<1 hour	Other	Called or attended at GP	No
P26	80	<1 hour	Other	Ambulance to A+E	No
P27	61	<1 hour	Other	Called or attended at GP	No
P29	87	<1 hour	Other	Called or attended at GP	No
P31	89	<1 hour	Other	Routine visit by nurse	No
P15	80	<1 hour	Self and other	Called or attended at GP	No
P23	75	<1 hour	Self and other	Called or attended at GP	Yes
P28	61	<1 hour	Self and other	Called or attended at GP	No
P34	52	<1 hour	Self and other	Called or attended at GP	Yes
P22	77	<1 hour	Self and other	Called or attended at GP	No
P32	73	<1 hour	Self	Called or attended at optician	No
P33	61	<1 hour	Self	Attended A+E	Yes
P18	80	<1 day	Self and other	Attended A+E	Yes
P24	85	<1 day	Self and other	Called or attended at GP	No
P19	65	<1 day	Self	Called or attended at optician	Suspected
P30	45	<1 day	Self	Called or attended at GP	Suspected
P16	73	>1 day	Self	Called or attended at GP	No
P17	72	>1 day	Self	Routine doctor visit	Suspected
P25	71	>1 day	Self	Called or attended at GP	No
P21	63	>1 day	Self and other	Called or attended at optician	No

### Symptom Recognition

Participants' actual symptoms and the symptoms they had associated with TIA or stroke prior to their own experience are described below and shown in Table 2. Participants varied in the extent to which they identified their symptoms as a TIA.

**Table 2:** Symptoms expected and experienced by participants.

Symptom Expected	%	Symptom Experienced	%
Loss of use of one side of body	25	Speech problem	60
Speech problem	20	Impaired use hands or arm	50
Face or mouth drop	20	Face or mouth drop	30
Impaired use hands or arm	20	Vision problems	30
Losing consciousness	20	Tingling or numb hands or arms	25
Falling over	15	Tingling or numb legs	15
Impaired use legs	15	Face or head pain	15
Overall tingling or numbing	10	Feeling funny or unwell	15
Face or head pain	10	Loss of balance	10
Unable to swallow	10	Overall tingling or numbing	10
Vision problems	10	Mindless or not quite with it	10
Chest pains	5	Falling over	5
Tingling or numb face	5	Hyperventilating	5
Feeling funny	5	Not walking straight	5
Inability to move	5	Loss of colour	5
Jaw stiffness	5		

*Note.* %, percentage of participants who expected or experienced each symptom.

#### Symptom Expectation and Experience

Although a variety of symptoms were mentioned, problems with speech were the most common symptoms both expected and experienced by participants. Face drop and impaired use of arms also featured regularly in participants' accounts of both expected and experienced symptoms. These three symptoms aside, symptoms were generally expected to be more dramatic or severe with participants associating loss of consciousness or falling down with a stroke event prior to their own experience. By contrast, many participants experienced milder more ambiguous symptoms such as vision problems, tingling or feeling unwell. There was some confusion in the differentiation between stroke and TIA; the existence of TIA and its more transient symptoms was a new discovery for some participants.

*I thought a stroke was a stroke and like you say you would be likely to be struck down rather than experience what I'd experienced.*

(P25, M71, self, >1 day delay)

*I thought it'd be more dramatic. You know, expect to be sort of almost conscious one minute and not the next sort of thing. That was the impression I had, that, you know, it was a falling over sort of problem … but I wasn't aware that there was a category where it can come and go so quickly, that was the thing that got me was the fact that it all happened within ten minutes and ten minutes later I was improving.*

(P27, M61, other, <1 hour delay)

#### TIA Identified

Four participants identified TIA based on recognition of key symptoms or previous stroke experience. Three of these participants contacted emergency services or enlisted the help of others to seek immediate medical attention. For one participant, recognising symptoms did not prompt immediate action. Despite correctly identifying symptoms based on a previous experience, this participant used her ability to speak as a reason to delay seeking care. It was only when her symptoms impacted on her life, through an inability to write, that she decided to take action.

*Because the first time I couldn't [speak], not intelligibly, so I could speak and I thought oh that's all right, I shall not bother anybody, but when I came to write my diary later in the day I couldn't, my right hand had gone sort of half numb and so after that I thought oh I had better go to the hospital, so I did.*

(P18, F80, stroke two years ago, self and other, <1 day delay)

#### TIA Suspected

Three participants suspected that they might have experienced a TIA but did not seek medical attention straight away. Care-seeking was delayed due to a perceived lack of severity, with the understanding that medical attention could be sought later if symptoms worsened.

Interviewer: *Did you think it was another TIA or?*

P30: *I thought it probably was, but it didn't seem too severe and I didn't have the headache…*

Interviewer: *Right, what made you think it was a TIA?*

P30: *Because, you know, they told me about symptoms at [hospital name], about what to look for and I thought, but it was only a tingling on one of the arms and I thought well, it probably isn't, it might be but it probably isn't. So I wasn't overly concerned. If it had got worse, I would have gone almost straight away anyway.*

(P30, F45, self, TIA 2 years ago, <1 day delay)

#### TIA Not Identified

Thirteen participants did not consider their symptoms to be indicative of TIA at the time. The absence of typical stroke symptoms, like face drop or speech problems, resulted in the dismissal of stroke or TIA as a possible cause leading to reluctance to seek medical care.

*I remembered that advert on the TV and I started, well I can do that you know I can lift my hands up and I had a look in the mirror when I was talking and my mouth didn't drop or anything.*

(P16, M73, self, >1 day delay)

When unexpected or ambiguous symptoms were experienced, participants described attributing symptoms to alternative causes such as eye problems which could lead to delays in seeking appropriate care.

*I went to see the opticians first; I thought something had gone up with my eyes which I would do with something like that. So phoned the opticians or my partner did, she phoned up to make an appointment.*

(P21, M63, self and other, stroke three years ago, >1 day delay)

Lack of TIA recognition also arose from impaired overall awareness. Participants expressed greater concern with the actual symptoms than on applying a label or trying to identify a cause. It was only later, when initial care had already been sought, that the possibility of TIA was considered.

P26: *Well, I mean, while it was happening, you know, I didn't think that. I was just shocked like what happened really.*

Interviewer: *But then afterwards did you think…?*

P26: *Well afterwards I thought that was a mild stroke of some sort like you know.*

(P26, M80, other, stroke 10 years ago, <1 hour delay)

Overall, patients' recognition or suspicion that symptoms may be indicative of stroke or TIA could lead to making urgent contact with medical services. This was not always the case however, and recognition of symptoms appeared to interact with other contextual factors in participants' descriptions of delay in contacting medical services. In addition, although the majority of participants did not identify TIA at the time, lack of recognition did not always result in delay, in particular when others were involved in decisions to seek care.

### Care-Seeker

Based on the systematic review by Sprigg and colleagues [15] an original category of presence of a witness was applied to data in the early stages of analysis. As analysis progressed it became apparent that simply exploring whether witnesses were present or not was not particularly informative and that participant accounts also provided details on whether the patients or others made initial contact with medical services. Consequently, this category was re-named as care-seeker and applied to all transcripts.

We defined the care-seeker as the person who made the decision to seek care and/or the person who enacted the care-seeking behaviours (e.g. phoning the GP surgery, driving to the hospital). In five cases others sought care on behalf of the patient, seven participants sought care for themselves and in eight cases care-seeking was a combined effort of the patient and friends or relatives.

#### Other as Care-seeker

For five participants, others were present at onset of symptoms and sought care on their behalf. In all five cases, medical services were contacted within an hour. For four of these participants, the care-seeker was a friend or family member. In one case, a community health nurse who was attending for a routine visit phoned emergency services.

Other people sought care for patients when symptoms that were experienced impacted on patients' ability to make their own care-seeking decisions. In these situations, participants were not fully aware of what was taking place and did not engage with the decision-making process. Participants described how others performed the action as their own attention was directed towards the symptom experience.

*It was people around me that did the action, not me. I was completely just, incensed that I couldn't speak and I couldn't focus on things and so, you know, at that time I didn't know what was happening completely. As I say, I just like so shocked at what was going wrong with me like, you know.*

(P26, M80, other, <1 hour)

None of these five participants were aware they were experiencing a TIA at the time. Three participants had not noticed any symptoms and indicated that if alone, they would not have sought urgent medical care. Those that did notice their symptoms described the likelihood that they would have attributed symptoms to an alternative cause.

*I was on the telephone to a friend, she's the one who instigated getting the doctor in… and she says you're not talking very well. I was as far as I knew…so she said I think you ought to get the doctor. I said oh you know don't bother. But anyway the doctor did come and she checked me out.*

(P29, F87, other, <1 hour)

*To be honest if I'd been on my own, I don't think I would have even sought any medical help, because I would have just put it down to oh it was cold, I would have put it down to oh perhaps it was just a blip in my blood pressure or any number of things.*

(P27, M61, other, <1 hour)

Two of these participants described how they did not want to seek medical attention but their opinions were overruled by those that sought care on their behalf. Participants' preferences appeared to have relatively little impact as decisions were made by others regardless of the patients' wishes.

*I said I ain't going to hospital and she [granddaughter] said you are and sent for the ambulance.*

(P20, M85, other, <1 hour)

#### Self as Care-seeker

Seven participants sought their own medical care following TIA. Two sought care within an hour, two sought care within a day while three took longer than a day to seek medical attention in response to their TIA symptoms. Participants elaborated on their reason for seeking medical attention during the interviews. Previous TIA experience, concordance between expected and experienced symptoms and feeling afraid because they were alone were mentioned as reasons for seeking medical attention as soon as possible. Those participants who did not seek immediate care described delaying care-seeking due to the mild transient nature of their symptoms.

Interviewer: *So why didn't you go immediately, was it because you didn't see it?*

P17: *No because it went away. It went away*

(P17, M72, self, >1 day delay)

Although these seven participants made and enacted their own care-seeking decisions, interactions with friends and family members could sometimes be incorporated into the decision making process. These interactions could both encourage or discourage care-seeking by undermining the severity of symptoms or recommending seeking medical attention.

*I was away for my hair cut at a friend's … and I felt a tingling in half of my lips you know just one side, I said “Can you see anything wrong with my face?” and of course she started laughing, she said “No, why?” I said “I feel a tingling sensation in it”, she said “You look fine” so I sat down and had a cup of tea and that and was there for ages talking and it went.*

(P16, M73, self, >1 day delay)

*So I mentioned it to somebody that I confided in and they said well I should go to the doctor. And I'd half thought that myself you see if that had happened again I would go but I thought I will go so I went straight down to the doctor.*

(P25, M71, self, >1 day delay)

#### Care-seeking as Collaboration

Eight participants described collaboration between themselves and friends or relatives in contacting medical services. Delay was less than an hour for five participants, less than a day for two participants and longer than a day for one participant. Three participants described contacting family or friend members not present at symptom onset to seek care on their behalf. These descriptions had much in common with the care-seeking enacted by others alone and participants appeared to leave responsibility for contacting medical services to the friends or family members initially contacted.

*I just pulled onto the side of the road and then I phoned my son and they come out and picked me up…anyway I got back here and I just said I just want to go to bed, have a sleep and my wife phoned the doctor.*

(P28, M61, self and other, <1 hour)

For the other five participants, relatives and friends played a more practical role in enacting participants' decisions to seek or delay contacting medical services. These friends and family members provided practical support by calling the doctor or driving the patient to the GP or hospital but did not have as great an influence on the decision to seek care. Similar to participants who sought care for themselves, some of these participants elaborated on the reasons for their delay.

Interviewer: *Was there any reason why you didn't seek help straight away?*

P21: *Get this shift out the way because I only had the one shift that week. That's all I'd got.*

(P21, M63, self and other, stroke three years ago, >1 day delay)

### Type of Care Sought

There was variation in the types of initial contact with medical services. Twelve participants called or attended their GP in response to TIA symptoms and a further two were diagnosed during routine visits. Of those that initially contacted their GP, three were instructed to call an ambulance and subsequently attended at the Emergency Department (ED). Two participants attended straight to the ED, and one called for an ambulance. The final three participants made initial contact with their optician; in one case the optician recommended attendance at the ED.

Of the participants who used emergency services, two did so within an hour and one did so within a few hours of symptom onset. Emergency services were contacted when participants or friends of family members identified a need for immediate assistance. One participant described driving straight to the ED to avoid delay as she presumed her GP would recommend attending at hospital.

*I thought if I go to the doctors they'll only take me to the hospital, so I got myself to the hospital even though it was very crowded and there was a huge traffic jam.*

(P33, F61, self, <1 hour delay)

GP contact was the most common type of care sought and there was a variation in length of delay among the 12 participants who called or attended at their GP. The option of contacting emergency services directly was not discussed by any of these participants even though contact was made with the GP within an hour of symptom onset in eight cases.

Participants who attended at the optician also varied in presentation delay with one making contact almost immediately, one calling the optician within a day and one delaying for longer than a day. These participants chose to contact their optician as they experienced vision problems and attributed their symptoms to problems with their eyes.

### Secondary Delays

Although the focus of the analysis was on the initial care sought, an additional theme of secondary delays was identified during the analysis where participants described delays between initial contact with medical services and attending at the hospital. Other commitments, such as pre-booked holidays or family events, could result in participants not attending at the soonest available hospital appointment.

*The appointment was for the next day but I couldn't go because I was going to Scotland for a family funeral so it was like a week before, but it was my fault and not theirs.*

(P16, M73, self, 2 week delay to hospital attendance)

Initial interactions with health care professionals could also impact on time until treatment was received. Participants that contacted emergency services, either straight away or on the recommendation of their GP, commented on the speed of paramedics' response.

*[My wife spoke to] the ambulance service and she says oh there's an ambulance on its way, the next thing two paramedics walked in. I couldn't believe how quick they got here.*

(P28, M61, self and other, <1 day delay to hospital attendance)

Conversely, health care interactions could also cause confusion over the importance of the event and prolong the time to hospital attendance. Delay resulted from practical issues such as lack of available appointments and from perceived low priority placed on the event by the healthcare professional initially contacted. One participant described a lack of urgency from his optician after experiencing a problem with his vision in his left eye. As a result, he delayed seeking further care and only mentioned the incident to his GP when attending at a routine appointment four weeks later.

Interviewer: *How quickly did you get from the opticians to, did you get, can't remember was it your GP or the hospital?*

P32: *Well I didn't bother, I didn't bother because he couldn't find anything and… I thought well, I mean this was sort of late November, early December, I always see him before Christmas anyway, and I'd got to see, I was going to see him anyway so I went probably, it was probably about four weeks you see that I'd lapsed.*

(P32, M73, self, TIA 8 years ago, 7 week delay to hospital attendance)

## Discussion

### Main Findings

Decisions to seek care following TIA are not simple and a number of elements combine to cause delay in seeking medical care. By focusing on patients' descriptions of decisions to seek care, the current study demonstrated the overlapping factors that contribute to real life care-seeking delays. Most participants did not recognise their experience as a TIA, sometimes due to the presence of ambiguous symptoms and the expectation of a more dramatic event. However, participants' recognition of symptoms was not always the driving force in care-seeking decisions. Friends or family members were involved in care-seeking for many participants, but the role of these friends or family members varied widely from deciding on and enacting care-seeking decisions to simply driving the patient to the GP practice. Finally, secondary delays, sometimes as a result of interactions with healthcare professionals, could result in long delays in accessing appropriate treatment even when initial care was sought quickly.

### Strengths and Limitations

A previous study of patients' behaviour immediately after TIA in the UK reported that 87% of patients first seek medical care from the GP, 10% use emergency services and 3% contact other sources [10] Although the sample in this study was not intended to be representative, the inclusion of patients in all three of these groups allowed care-seeking delay to be explored across patients with varied experiences. Similarly, our study included descriptions of care sought by patients themselves, friends and relatives or a combination of the two to provide a fuller understanding of care-seeking following TIA in real life contexts.

Our findings should be viewed in the context of the sampling limitations of the current study. As with previous qualitative studies in TIA populations (e.g. [18]) participation in this study was limited to patients who did eventually seek care and had received a TIA diagnosis. Consequently, participants' recall of symptoms and care-seeking decisions may have been affected by the subsequent attachment of a TIA label to their experiences. Previous research has indicated that many people experience symptoms consistent with a TIA but never seek medical attention [4] and our recruitment strategy precluded the inclusion of the experiences of these patients in our study. Future research with such a sample would be an interesting addition to the care-seeking literature although identifying and recruiting patients who have never received a TIA diagnosis remains a challenge.

Our response rate was similar to reported rates in other qualitative studies conducted with TIA patients [18]. Although, as in all research studies, it is possible that patients who choose to participate may differ from those that don't, participants in the current study did not differ in terms of gender and postcode index of multiple deprivation scores from those that did not take part.

The retrospective nature of patients' accounts and the potential for cognitive impairment following TIA introduce the possibility of inaccurate recall. All participants were deemed competent to take part in the interviews by health care professionals and overall appeared to give coherent accounts of their experience from their own perspective. Confusion or uncertainty did form part of some participants' descriptions of their TIA experiences, but as the aim of the current study was patients' perspectives of care-seeking, rather than to elicit accurate symptom reports, these experiences were deemed as valid as any others.

Unlike previous qualitative studies with stroke patients [16], [17], we did not adopt a particular theoretical framework for analysis. Given the lack of robust theoretical evidence on care-seeking following TIA we decided not to attempt to fit the data to existing theories or models. Our results do offer some interesting avenues for future theoretical development however, especially in relation to the impact of stroke and TIA on cognitive functioning. It is clear in our interviews that some patients struggled with appraising symptoms as indicating illness, a core features of models of help-seeking delay, as their capacity to recognise their symptoms was impaired [12], [13]. Probably more important in the present sample though, was the role of others in making and enacting care-seeking decisions. Our findings suggest there may be a need to place greater emphasis on the collaboration between patients and important others in care-seeking decisions for conditions such as stroke or TIA where family and friends are likely to play a central role.

### Relationship to Past Literature

The factors identified by Sprigg and colleagues [15] in their systematic review of the quantitative evidence of delay in seeking medical attention following TIA provided a useful framework for the current analysis. By using patient narratives to explore delay our findings highlight the different ways in which factors including symptom recognition, the presence of others and type of care sought can interact for individual patients. For example, the inconsistencies between patients' recognition of TIA symptoms and care-seeking identified by Sprigg and colleagues [15] in the quantitative literature may reflect the different ways in which patients and friends and family can influence decisions to seek medical care and the relative importance placed on symptoms by patients.

Our findings also extends on previous research of type of care sought which demonstrated that patients with TIA are less likely to contact emergency services than patients with stroke [15]. All patients included in the current study had sought care and yet most did not report even considering the use of emergency medical services. In addition, three participants described initially attending at their optician due to visual impairment. The role of the optician in TIA diagnosis and subsequent treatment has not previously been explored.

Our study extends existing qualitative studies of patient experiences of TIA [19] by focusing on care-seeking and explicitly asking participants to describe expected symptoms and comparing these with the TIA symptoms actually experienced. While speech problems, face drop and inability to use arms were both expected and experienced, the presence of less typical symptoms, such as tingling, pain and feeling unwell, was also common. Vision problems were the main symptoms described by a number of participants and led to initial contact being made with the optician in some cases. In line with previous research, vision problems were poorly recognised as indicative of a TIA or stroke event [23].

Previous research has indicated that patients experiencing symptoms associated with a higher risk of subsequent stroke, for example motor symptoms, seek care more quickly [10]. Our analysis suggests this may be due to the reduced influence of the patient on care-seeking decision in these cases resulting in the swifter action more typically associated with the behaviour of friends or family members. Our study also demonstrated that even the correct recognition of TIA is not a guarantee that a patient will immediately contact medical services and that other factors, such as the impact of symptoms on functioning, may also be incorporated into patients' decisions to seek care.

Similar to previous research with stroke patients our study demonstrated the important role played by others in care-seeking delays in response to TIA symptoms [16], [17]. Mackintosh et al. (2012) identified a number of purposes for contacting others following stroke including taking responsibility for interaction with medical services, affirmation and comfort and reassurance. Our study elaborated on the impact of others in care-seeking delay by identifying a continuum of influence ranging from full responsibility, sometimes against the wishes of the patient themselves, to simply enacting the behaviours desired by the patient. This finding may be more relevant to TIA than stroke as the transient nature of TIA symptoms may increase the likelihood that patients themselves contribute to decisions to seek medical care.

### Implications for Research and Practice

Our study indicated that while patients who seek care are aware of the serious symptoms typically associated with stroke there is less awareness of the milder more transient symptoms also common in TIA. The presence of less severe, unexpected or atypical symptoms has been shown to be associated with delays in contacting medical services in cancer and myocardial infarction patients [14], [24]. Future work can build on the current study to investigate the importance of symptom expectation and the presence of atypical symptoms in explaining delays in receiving appropriate medical attention following TIA. In addition, there may be a need in future public health campaigns to emphasise that while common symptoms can help identify TIA or stroke not all symptoms will be experienced in all cases and that the absence of typical symptoms is not a guarantee that no cerebrovascular incident has taken place.

Given the qualitative nature of the current study, the hierarchy of importance of individual factors in explaining delay in contacting medical services could not be explored. However, the role of others in care-seeking was referred to across the other domains described by participants. Consequently, there may be reason to explore additional factors, including symptom recognition, with reference to the care-seeker. Future research should focus not only on the individual who contacts medical services but also on the role of others in how decisions to seek care are made.

Most participants in the current study made initial contact with their GP or optician demonstrating the potential important role of these health care professionals for patients who do seek care. As the first line of contact for many patients, GPs, opticians and their reception staff should be trained to emphasise the importance of immediate action in response to TIA symptoms and facilitate patients in accessing urgent appropriate treatment. Future intervention strategies should reinforce calling for an ambulance in response to a suspected TIA given that using emergency services has the most consistent association with reduced care-seeking delay but was not even considered by the majority of participants in this study.

### Conclusions

As the first qualitative exploration of delay in contacting medical services following TIA, this study identified a number of over-lapping factors that can contribute to delays in seeking medical help. The study highlights the need to increase awareness of the milder more transient symptoms that can be associated with TIA and suggests avenues for future research into the specific role played by friends and family members in making and enacting care-seeking decisions. The study also suggests that improving uptake of appropriate treatment following TIA does not end with the first contact with medical services and that initial healthcare interactions are an opportunity to emphasise the importance of subsequent attendance at a hospital clinic to patients.

## Supporting Information

Appendix S1 **Patient Consent Form.**
(DOCX)

Appendix S2 **Interview Guide.**
(DOCX)
