# Supplementary material for: Delay in Seeking Medical Help following Transient Ischemic Attack (TIA) or “Mini-Stroke”: A Qualitative Study
Source: PLoS One. 2014 Aug 19;9(8):e104434. doi: 10.1371/journal.pone.0104434 (PMC4138063; doi:10.1371/journal.pone.0104434)
Supplement: Appendix S1 — Patient Consent Form. (DOCX) [file pone.0104434.s001.docx]

**
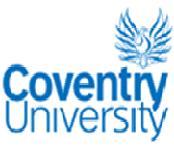
**

**Appendix S1. Patient Consent Form**

**Title of Project**: Developing awareness of Transient Ischaemic Attack (TIA) and behaviour change interventions for lifestyle intervention in primary care

**Lead Researcher**: Professor Louise Wallace

**Please initial box**

| I confirm that I have read and understood the Information Sheet for the above study and have had the opportunity to ask questions. |  |  |
| --- | --- | --- |
|  |  |  |
| I understand that my participation is voluntary and that I am free to withdraw at any time, without giving any reason. |  |  |
|  |  |  |
| I agree to take part in the first structured interview |  |  |
|  |  |  |
| I agree to take part in the second structured interview |  |  |
| I agree to the interviews being tape recorded |  |  |
| I agree to take part in a more in-depth interview in six months |  |  |
|  |  |  |
| I agree to you contacting my GP |  |  |
|  |  |  |
| I agree to my medical records being accessed by the  research team |  |  |
|  |  |  |
| I agree to take part in the study |  |  |
|  |  |  |

**GP Contact details**

Name of GP:

Surgery Address:

Telephone Number:

|  |  | |  |
| --- | --- | --- | --- |
| Signature of participant | |  | Date |
