# Supplementary material for: Delay in Seeking Medical Help following Transient Ischemic Attack (TIA) or “Mini-Stroke”: A Qualitative Study
Source: PLoS One. 2014 Aug 19;9(8):e104434. doi: 10.1371/journal.pone.0104434 (PMC4138063; doi:10.1371/journal.pone.0104434)
Supplement: Appendix S2 — Interview Guide. (DOCX) [file pone.0104434.s002.docx]

**Appendix S2. Interview Guide**

SECTION 1 - WHAT HAPPENED/SEEKING MEDICAL HELP

1) Please can you tell about your recent illness that led to you being seen by your GP/at the hospital?

Elicit story and use prompts:

- What symptoms did you experience?
- How quickly after having your first symptoms did you seek help?
- What did you do to seek help? (*Prompt: Called GP/999?*)
- If you did not seek help why not?

2) What did you think was happening to you?

3) What did health professionals say was happening to you?

(*Prompt: Did they say it was a TIA?)*

SECTION 2: PRE-CONCEPTIONS REGARDING TIA

1. What do you know about strokes?
2. Prior to being ill recently did you have any thoughts about what the symptoms of a TIA or ‘mini stroke’ might be?
3. Were the symptoms you thought you might have experienced before having a TIA the same or different to those symptoms you actually experienced when you were ill recently?
4. Have you seen the FAST advert on TV?

- How similar were the symptoms you experienced to those shown in the FAST advert/leaflet?
- To what extent did information provided in this advert influence the way in which you sought help?

1. Before your recent illness would you have had any ideas of how long symptoms or the affect of having had a TIA might last for? (ask for estimate in days/weeks/months)

- In what way(s) would you have thought that a TIA might have affected someone’s quality of life?

(*Prompt: What changes might they have had to make to the life they led before?)*

1. Before your recent illness, to what extent might you have thought someone could control whether they had a TIA or not?

- How much might you have thought medication could help someone control their experience of TIA?

*That completes this interview, thank you for taking the time to share your experience of having a TIA with me.*
